# Regulation of Endothelial Progenitor Cell Functions in Ischemic Heart Disease: New Therapeutic Targets for Cardiac Remodeling and Repair

**DOI:** 10.3389/fcvm.2022.896782

**Published:** 2022-05-23

**Authors:** Huai Huang, Weiqiang Huang

**Affiliations:** Department of Geriatric Cardiology, Guangxi Key Laboratory Base of Precision Medicine in Cardio-Cerebrovascular Diseases Control and Prevention, Guangxi Clinical Research Center for Cardio-Cerebrovascular Diseases, The First Affiliated Hospital of Guangxi Medical University, Nanning, China

**Keywords:** ischemic heart disease, endothelial progenitor cells, cardiac remodeling, cardiac repair, angiogenesis, crosstalk

## Abstract

Ischemic heart disease (IHD) is the leading cause of morbidity and mortality worldwide. Ischemia and hypoxia following myocardial infarction (MI) cause subsequent cardiomyocyte (CM) loss, cardiac remodeling, and heart failure. Endothelial progenitor cells (EPCs) are involved in vasculogenesis, angiogenesis and paracrine effects and thus have important clinical value in alternative processes for repairing damaged hearts. In fact, this study showed that the endogenous repair of EPCs may not be limited to a single cell type. EPC interactions with cardiac cell populations and mesenchymal stem cells (MSCs) in ischemic heart disease can attenuate cardiac inflammation and oxidative stress in a microenvironment, regulate cell survival and apoptosis, nourish CMs, enhance mature neovascularization, alleviate adverse ventricular remodeling after infarction and enhance ventricular function. In this review, we introduce the definition and discuss the origin and biological characteristics of EPCs and summarize the mechanisms of EPC recruitment in ischemic heart disease. We focus on the crosstalk between EPCs and endothelial cells (ECs), smooth muscle cells (SMCs), CMs, cardiac fibroblasts (CFs), cardiac progenitor cells (CPCs), and MSCs during cardiac remodeling and repair. Finally, we discuss the translation of EPC therapy to the clinic and treatment strategies.

## Introduction

Cardiovascular disease (CVD), primarily ischemic heart disease (IHD), remains the leading cause of global disability and mortality (1). IHD is characterized by a loss of myocardial cell function due to insufficient blood supply flowing from the coronary arteries to the heart, followed by ventricular dysfunction and progressive heart failure. After myocardial infarction (MI), tissue damage and necrosis initiate a series of pathological responses, such as inflammation, oxidative stress, neurohormonal system activation, and hemodynamic load regulation, which lead to fibrotic scarring during the replacement of lost cardiomyocytes (CMs) (2, 3). Subsequently, the injured heart undergoes remodeling which leads to further cardiac hypertrophy, ventricular dilation and reduced contractility involving cellular and interstitial changes at the myocardial tissue level, mainly fibrosis, which ultimately leads to fatal heart failure (4). The current treatment for IHD mainly involves percutaneous coronary intervention (PCI) and coronary artery bypass grafting (CABG) to achieve revascularization, increase blood supply and save the injured ischemic myocardium (5).

Stem cell therapy has been widely used to treat IHD, providing patients with drug-free and non-surgical treatment and encouraging safer and more feasible cardiac repair strategies (6). Endothelial progenitor cells (EPCs), also known as vascular endothelial precursor cells, exhibit the potential to enter the blood circulation and undergo proliferation and differentiation into multiple cell types (7). Studies have shown that EPC number and function are closely related to endothelial cell (EC) injury and dysfunction and can be used in the clinic as biomarkers of vascular function and cumulative cardiovascular risk (8). EPCs are involved in cardiac repair after MI through regulation of immune responses, neovascularization, extracellular matrix deposition and cardiac microenvironment formation. In this review, we introduce the biological characteristics and recruitment mechanism of EPCs, discuss the impact of crosstalk between EPCs and related cardiac cells and mesenchymal stem cells (MSCs) on cardiac remodeling and repair after ischemic cardiomyopathy, and report the current clinical implications of EPC therapy.

## The Definition, Origin, and Biological Significance of EPCS

In 1997, Asahara and colleagues were the first researchers to identify a population of cells capable of differentiating into mature ECs and undergoing postnatal angiogenesis, and they called the cells in this subset “putative endothelial progenitor cells” (9). It was initially thought that EPCs originated and resided in bone marrow (BM) and were mobilized to circulate in adult peripheral blood (PB) or umbilical cord blood (UCB) (10). Since no uniform isolation or culturing protocol for EPCs has been reported and because EPCs express different surface antigens at different stages of maturation or differentiation, EPC subpopulations exhibit various phenotypes (11). Two clear types of EPC have been identified on the basis phenotype and function: hematopoietic EPCs (myeloid) and endothelial lineage EPCs (12). The hematopoietic EPC lineage includes myeloid angiogenic cells (MACs), circulating angiogenic cells (CACs) and early EPCs. EPCs with this immunophenotype express CD45, CD14, and CD31 but not CD146 or CD133. These cells do not have the capacity to differentiate into ECs but promote angiogenesis through paracrine signaling. The endothelial lineage includes endothelial outgrowth cells (OECs) or late EPCs and endothelial colony-forming cells (ECFCs), which are also widely recognized as “bona fide EPCs” and show the ability to undergo clonal expansion and self-renewal, form blood vessels and intima, and continuously be incorporate into and contribute to the formation of the host vascular system (12–14). Recently, CD133, CD34, and VEGFR-2 (also known as KDR or Flk-1) have been the most commonly used biomarkers to identify or characterize EPCs (15). However, other recent studies have shown that circulating CD19^−^ CD34^+^ EPCs do not express VEGFR-2, which is expressed only in CD19+ B cells (16). When expression of sca1+/flk1+ cells were observed in mice, the expression of B-cell-specific surface markers was found to be upregulated. It has been speculated that currently characterized EPCs are not truly EPCs but are lymphocytes, mainly B2 lymphocytes; therefore, scientists need to engage in further research and discussion (17). Although BM is considered the classic source of EPCs, Fujisawa et al. found that circulating EPCs isolated from vessel walls and PB of male patients who had undergone BM transplantation from female donors displayed XY genotype (18). In addition, Ingram et al. found that a complete hierarchy of EPCs can be identified in human umbilical vein ECs or human aortic ECs, showing that EPCs may originate from blood vessels (19). EPCs have been found to reside in the vasculature beds of various tissues, and CD157 or EPCR have been proposed as markers to identify tissue-resident EPCs (20). Surprisingly, a recent study reported marker genes, secreted factors, microRNAs (miRNAs), and growth factors of EPCs on the basis of single-cell transcriptomic analyses to better optimize and characterize the EPC subpopulation in adult PB. In this study, BMB2, BMP4, and Ephrin B2 were highly expressed only in EPCs, not ECs, in three different tissues. The neuropilin-1, VEGF-C, Notch 1, PECAM-1, and MIR-21 genes were differentially expressed. CD62L and PLAUR expression levels can be used as markers for the isolation and characterization of EPCs derived from monocytes (21).

EPCs participate in vascular and cardiac repair in coronary atherosclerosis (22), ischemic cardiomyopathy (23), and diabetic cardiomyopathy (24). During tissue or vascular injury, circulating EPCs are recruited to injury sites, enabling the growth of new blood vessels that are formed from an extension of existing ECs (angiogenesis) or *de novo* (vasculogenesis) (25). At the same time, soluble factors and EPC exosomes (EXs) and microvesicles (MVs) secreted by EPCs can attenuate a deleterious microenvironment formed during inflammation and oxidative stress (26), regulate cell survival and apoptosis (27), inhibit mesenchymal cell transformation and fibrosis (28), promote the homing of stem/progenitor cells (29), nourish CMs and induce angiogenesis (30), thereby exerting beneficial effects.

## Recruitment Mechanism of EPCS in Ischemic Heart Disease

**Figure 1:**
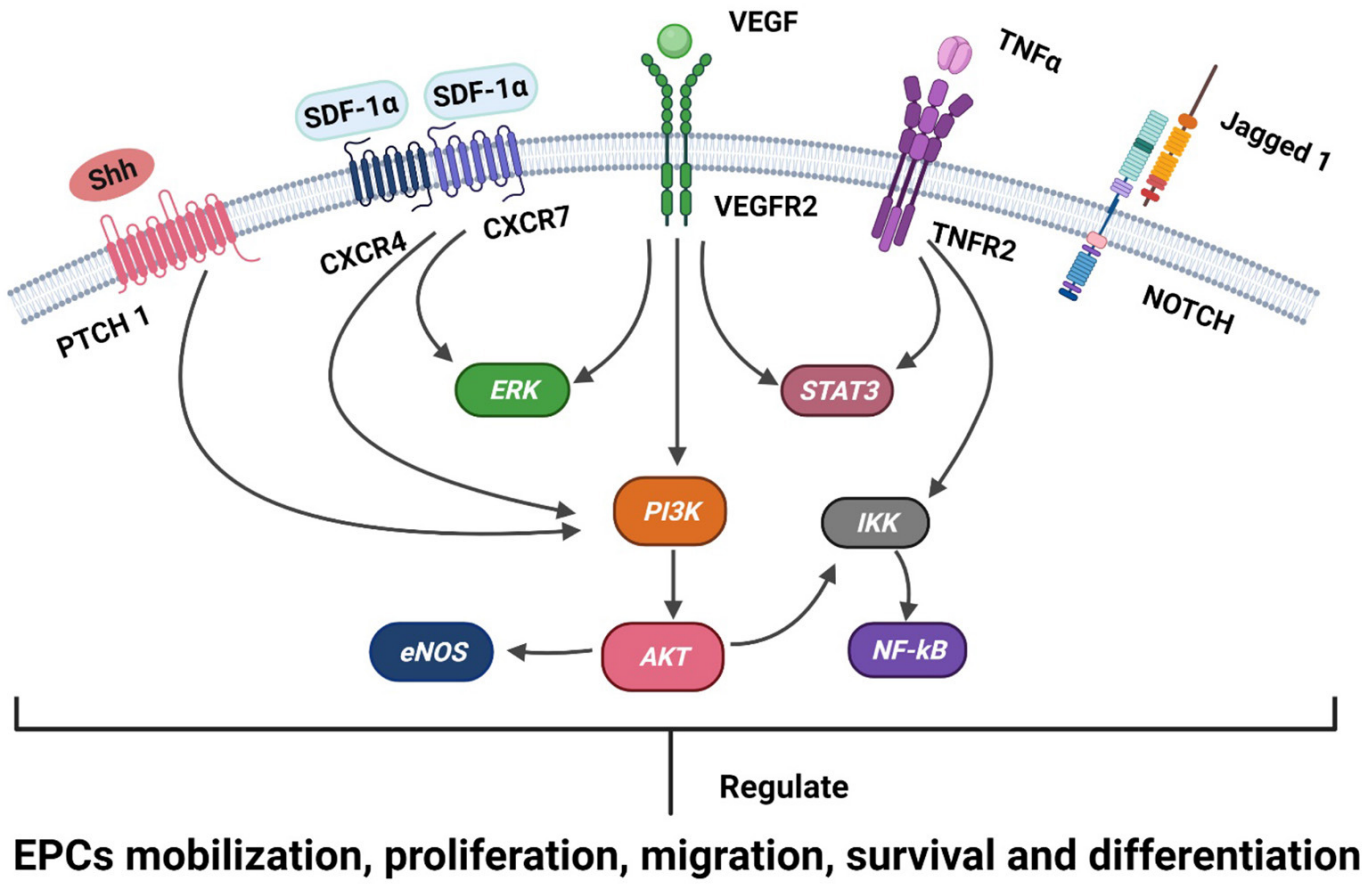
A schematic representation of the known signaling mechanisms of EPC recruitment in ischemic heart disease is shown. Receptors (Patch, CXCR4, CXCR7, VEGFR2, TNFR2, and Notch) and ligands (SHH, SDF-1, VEGF, TNFα, and Jagged 1) are shown on the membranes of EPCs. The SDF-1α/CXCR4/CXCR7, VEGFR, TNFα, SHH, and Notch signaling pathways engage in crosstalk with the intracellular PI3K/Akt/eNOS, ERK1/2, STAT3, and NF-kB pathways and regulate the development of EPCs in ischemic heart disease, including the mobilization, proliferation, migration, survival, and differentiation of EPCs.

EPCs are recruited from BM to ischemic tissues, and the processes (mobilization, proliferation, migration, and differentiation) that mediate neovascularization and re-endothelialization involve the regulation of multiple cytokines, receptors, adhesion molecules, proteases, and cell signaling mechanisms (31, 32) (Figure 1).

## EPC Recruitment is Regulated by Non-Coding RNAs

Non-coding RNAs (ncRNAs) constitute classes of genetic, epigenetic and translational regulators, including miRNAs, long ncRNAs (lncRNAs), and circular RNAs (circRNAs), which play important roles in the development of CVD (33). ncRNAs were also found to be involved in the biological activities of endothelial progenitor cells (EPCs), including their mobilization, proliferation, migration, invasion, angiogenesis, and differentiation (Table 1).

**Table 1:** Non-coding RNAs regulate EPCs recruitment.

**Non-coding RNA**	**Positive/Negative**	**Targets**	**Function**	**Reference**
MicroRNA
miR-130a	+	VEGFR2/STAT3/HIF1α	Promotes EPCs proliferation, migration and tube formation under hypoxia	(34)
miR-210-3p	+	RGMA	Promotes EPCs proliferation, migration and tube formation under oxygen-glucose deprivation	(35)
miR-326-5p	+	Wnt1	Promotes angiogenesis ability of EPCs *in vitro* and *in vivo*	(36)
miR-126	+	Notch 1	Promotes EPCs proliferation, migration and invasion	(37)
	+	PI3K/Akt/GSK3β and ERK1/2	Promotes EPCs proliferation, migration and tube formation under oxidative stress	(38)
miR-324-5p	+	Mtfr1	Promotes the survival and proliferation of EPCs under oxidative stress	(39)
miR-144-3p	–	Ets1/MMP9	Impairs mobilization of EPCs	(40)
**lncRNA**
LncRNA WTAPP1	+	miR-3120-5P/PI3K/Akt/mTOR	Promotes migration, invasion, and tube-forming ability of EPCs *in vitro* and *in vivo*	(41)
lncRNA-p21	+	SESN2/AMPK/TSC	Promotes adhesion and migration of EPCs alleviates AngII-induced senescence	(42)
TUG1	+	miR-6321/ ATF2	Promotes EPCs migration, invasion and differentiation	(43)
	+	miR-29c-3p/PDGF-BB/Wnt	Promotes high glucose-impaired EPCs migration, invasion and tube formation	(44)
MALAT1	–	miR-15b-5p/MAPK1and mTOR	Inhibits EPCs proliferation and autophagy	(22)
THRIL	–	AKT pathway and FUS protein	Inhibits the proliferation of EPCs	(45)
circ RNA				
mmu_circ_0000250	+	miR-128-3p/SIRT1	Promotes high glucose-induced EPCs survival and angiogenesis	(46)
hsa_circ_0058092	+	miR-217/FOXO3	Promotes high glucose-induced EPCs survival, proliferation, migration and angiogenic differentiation	(47)
circ-ADAM9	–	mir-20a-5p/PTEN andATG7	Inhibits high glucose-induced EPCs survival and angiogenic differentiation	(48)

MiRNAs are endogenous small non-coding single-stranded RNAs with regulatory activity that lead to degradation of target mRNA or reduced protein translation by binding to the complete or incomplete complementary site of the 3'-untranslated region (UTR) of target mRNA (49). Chang et al. identified the expression profiles of miRNAs in early EPCs, late EPCs, and human umbilical vein ECs, and this profile can be used to determine novel biomarkers for the prevention and treatment of coronary artery disease (CAD) (50). Previous reviews have presented summaries of the roles played by miRNAs in regulating EPC proliferation, mobilization, migration, differentiation, apoptosis, autophagy, senescence, adhesion, and tubule formation, as well as EPC-induced angiogenesis (51). EPCs face the challenge of hypoxia in ischemic tissue, and overexpression of miR-130a and miR-210-3p at the cellular level can enhance the proliferation, migration and tube formation of EPCs under hypoxic conditions (34, 35). Li et al. found that EPCs transfected with miR-326-5p exhibited significantly increased tube formation in Matrigel plugs and angiogenesis in the MI model (36). Liu et al. observed that miR-144-3p in circulating exosomes disrupted the MMP-9 pathway by targeting the expression of Ets1 in MSCs and inhibited the mobilization of EPCs after myocardial infarction (40). Notably, recent studies have revealed that overexpression of miR-324-5p and miR-126 in EPCs can reduce the apoptosis and reactive oxygen species (ROS) production rates and prevent oxidative stress-induced EPC damage (38, 39). Maintaining the cardiac microenvironment by regulating oxidative stress can maintain endothelial stability and promote angiogenesis and remodeling.

LncRNAs are longer than 200 bp and exhibit regulatory ability but lack protein-coding capacity, and they play important roles in physiology and disease pathology (52). Li et al. found that the lncRNA WTAPP1 positively regulated the migration, invasion and angiogenesis of human peripheral blood-derived EPCs and mediated the effects of EPCs through the PI3K/Akt and autophagy pathways (41). By knocking down lncRNA-p21 expression, the adhesion and migration of EPCs were weakened, and the repairing effect of EPCs after vascular endothelial injury was disrupted (42). Overexpression of the lncRNA TUG1 promoted the migration and differentiation of EPCs and participated in angiogenesis (43, 44). The lncRNA MALAT1 inhibited EPC autophagy and apoptosis and increased cell viability by activating the mTOR signaling pathway, thereby delaying CAD progression (22). Xiao et al. found that lncRNA THRIL expression was upregulated in coronary atherosclerotic heart disease, and the lncRNA THRIL inhibited EPC proliferation and mediated autophagy through the AKT pathway and FUS protein activation (45).

circRNAs are non-coding RNAs in which the upstream 3' end is linked to the downstream 5' ends to form a closed-loop structure (53). circRNAs can regulate gene expression by regulating transcription and alternative splicing, interacting with RNA-binding proteins, or acting as miRNA sponges (54). Three studies revealed that mmu_circ_0000250 (46), and hsa_circ_0058092 (47) circ-ADAM9 (48) targeted their corresponding miRNAs, regulated autophagy and apoptosis of EPCs after treatment with high concentrations of glucose, and protected the migration and angiogenesis capacities of these EPCs. Hence, circRNAs have been suggested to interfere with EPC biological functions by absorbing miRNAs. However, studies on circRNA-mediated regulation of EPC functions in IHD have not been reported, and further exploration is needed.

## EPC Recruitment-Related Signaling Pathways

### The SDF-1α/CXCR4/CXCR7 Signaling Pathway

Stromal cell-derived factor-1alpha (SDF-1α) expression is increased in MI areas, while SDF-1α is required to stimulate stem/progenitor cell migration and homing to ischemic sites (55). Upon ligation with one of two receptors of the ligand SDF-1α (also known as CXCL12), CXCR4 and CXCR7, SDF-1α regulates the adhesion, transendothelial migration, proliferation, and tube formation of EPCs; specifically, EPC chemotaxis is mediated through only CXCR4, while EPC survival is mediated through the interaction of SDF-1α with CXCR7 but not that with CXCR4 (56). The CXCR7 expression levels were downregulated simultaneously in mouse myocardial tissue and EPCs after acute MI (AMI), and CXCR7 overexpression was found to rescue EPC migratory and angiogenic capacities (57). Qiu et al. found that SDF-1α expression upregulation and coupling of SDF-1α to the receptor CXCR4 contributed to anti-inflammatory and antiapoptotic effects after AMI (58). In addition, Fan et al. found that IL-1β promoted the EPC formation of capillaries and tubes in a CXCR7-dependent manner under inflammatory conditions and antagonized CXCR7 inhibition of EPC angiogenesis (59). The SDF-1α/CXCR4/CXCR7 pathway activates multiple signaling pathways and regulates multiple biological processes in EPCs. For example, the PI3K/Akt/ nitric oxide synthase (eNOS) pathway played a key role in stem/progenitor cell recruitment and angiogenesis, and SDF-1α-treated EPCs exhibited increased Akt and eNOS phosphorylation and nitric oxide (NO) production. However, PI3K inhibitor-induced Akt phosphorylation and eNOS expression were observed; thus, PI3K/Akt/eNOS pathway-mediated EPC apoptosis was inhibited (60–63). ERK participates in molecular signaling pathways involved in cell survival, differentiation and proliferation, and the ERK signaling pathway is involved in CXCR4- or CXCR7-mediated EPC proliferation, migration and angiogenesis (64, 65).

### The Vascular Endothelial Growth Factor Receptor Signaling Pathway

The mobilization, recruitment, and differentiation of EPCs and ECs are regulated by VEGF (66). VEGF increases the number of circulating EPCs and regulates *in situ* differentiation of EPCs and EPC formation of capillary plexuses (67). Hoffmann et al. used through high-throughput signaling pathway identification technology to examine the effect of VEGF signaling on BM-EPCs in the vasculature under hypoxia and found that VEGF-A-mediated VEGFR signaling was increased during hypoxia, while VEGF-A expression and VEGFR1 and VEGFR2 protein pathway activation was significantly increased (68). Interestingly, VEGF-A pathway activation in EPCs during hypoxia, while increases in related proteins were detected from NOS pathway, inositol and calcium signaling, G protein signaling, inflammation, and phospholipase signaling (68). In addition, VEGF has been shown to bind to VEGFR2 and activate the PI3K/Akt/eNOS (69), ERK1/2 (70, 71), and STAT3 pathways (34, 72). The VEGF signaling pathway in EPCs plays important potential roles in the regulation of redox homeostasis, cell survival, cell migration, angiogenesis, and vascular regeneration (68).

### TNFα Signaling Pathway

The TNFα/TNFR1 axis mediates post-MI cardiac dysfunction, while the TNFα/TNFR2 axis activation confers protection to ischemic hearts (73). TNFα binding to TNFR2 on EPCs activates the NF-kB signaling pathway to induce increased EPC migration *in vitro* (74). The TNFα/TNFR2 axis *in vivo* has been shown to be critical for ECFC survival, mobilization, and differentiation; VEGF expression; and ischemia-induced neovascularization (75). Naserian et al. found that EPCs modulated T-cell proliferation and acquisition of the proinflammatory phenotype, TNFα enhanced the immunomodulatory effect of ECFCs in an inflammatory environment, and the TNFα/TNFR2 signaling pathway enhanced the production of the anti-inflammatory cytokines TGFβ, IL-10, and HLA-G (76). In EPCs stimulated with TNFα, activation of the STAT3 signaling pathway through IL-10 overexpression enhanced EPC migration, adhesion, and tubule formation (77). These findings suggest that the TNFα/TNFR2 signaling pathway promotes the proliferation and migration of EPCs and has a cardioprotective effect in ischemic heart injury and MI.

### The Sonic Hedgehog Signaling Pathway

The Sonic Hedgehog (SHH) signaling pathway is a key regulator of postnatal angiogenesis and plays an important role in maintaining vascular homeostasis and angiogenesis (78). The Shh protein can stimulate BM-EPC proliferation and migration and VEGF production, which may promote angiogenesis in ischemic tissues (79). High-throughput RNA-sequencing and semiquantitative proteomic analysis have revealed that Hedgehog-interacting protein (HIP) expression was upregulated in late EPCs and inhibited hedgehog signaling. Activation of the Shh pathway after HIP expression downregulation during angiogenesis and oxidative stress enhanced angiogenesis and the function of newly sprouted aorta consisting of late EPCs (80). The Shh pathway was activated under hypoxia and oxidative stress, and the delivery of Shh protein enhanced EPC survival, migration, and tube formation (81). Carlos et al. found that microparticles carrying sonic hedgehog morphogen (MPShh+) significantly increased the expression of Shh signaling pathway genes and proangiogenic genes in EPCs, while Shh pathway-induced PI3K activation increased eNOS protein expression and activity, resulting in increased NO production. Most importantly, MPShh+ increased the angiogenic capacity of *in vitro* cultured EPCs of AMI patients to levels similar to those of healthy patients (82).

### The Notch Signaling Pathway

Notch signaling is involved in vascular development and affects EPC function in the BM microenvironment (83). Mammals express four Notch receptors (Notch 1 to 4) and five Notch ligands (Delta-like 1, 3, 4 and Jagged 1, 2) (84). EPC proliferation, migration and differentiation in the BM niche was stimulated by Jagged 1, but not Delta-like 1, contributing to post-ischemia angiogenesis (85). The regulatory mechanism of EPC homing and angiogenesis has been linked to the Notch pathway (37). Transforming growth factor-β-inducible protein-stimulated EPCs activated the NF-kB signaling pathway, inducing the expression of Notch ligands (delta-like 1 and Jagged1). Simultaneous activation of the Notch signaling pathway in adjacent EPCs stimulated the differentiation of EPCs into ECs (86). Guo et al. overexpressed hNotch1. The ICN gene in EPCs activated the Notch 1 signaling pathway and downstream effector molecules Hes1 and Hey1, enhancing the ability of EPCs to adhere to the endothelium, migrate across the endothelium, and form tubes (87). Li et al. studied a MI model and found that deer antler activated the Notch signaling pathway in EPC, upregulated the protein expression of Jagged 1, Notch1, NICD and HES1 and the mRNA expression of Hes1 and Hey2; it also promoted the mobilization of EPCs, endothelial repair and angiogenesis after MI (88).

### Crosstalk Between EPCs and Cardiac Cells During Cardiac Remodeling and Repair

**Figure 2:**
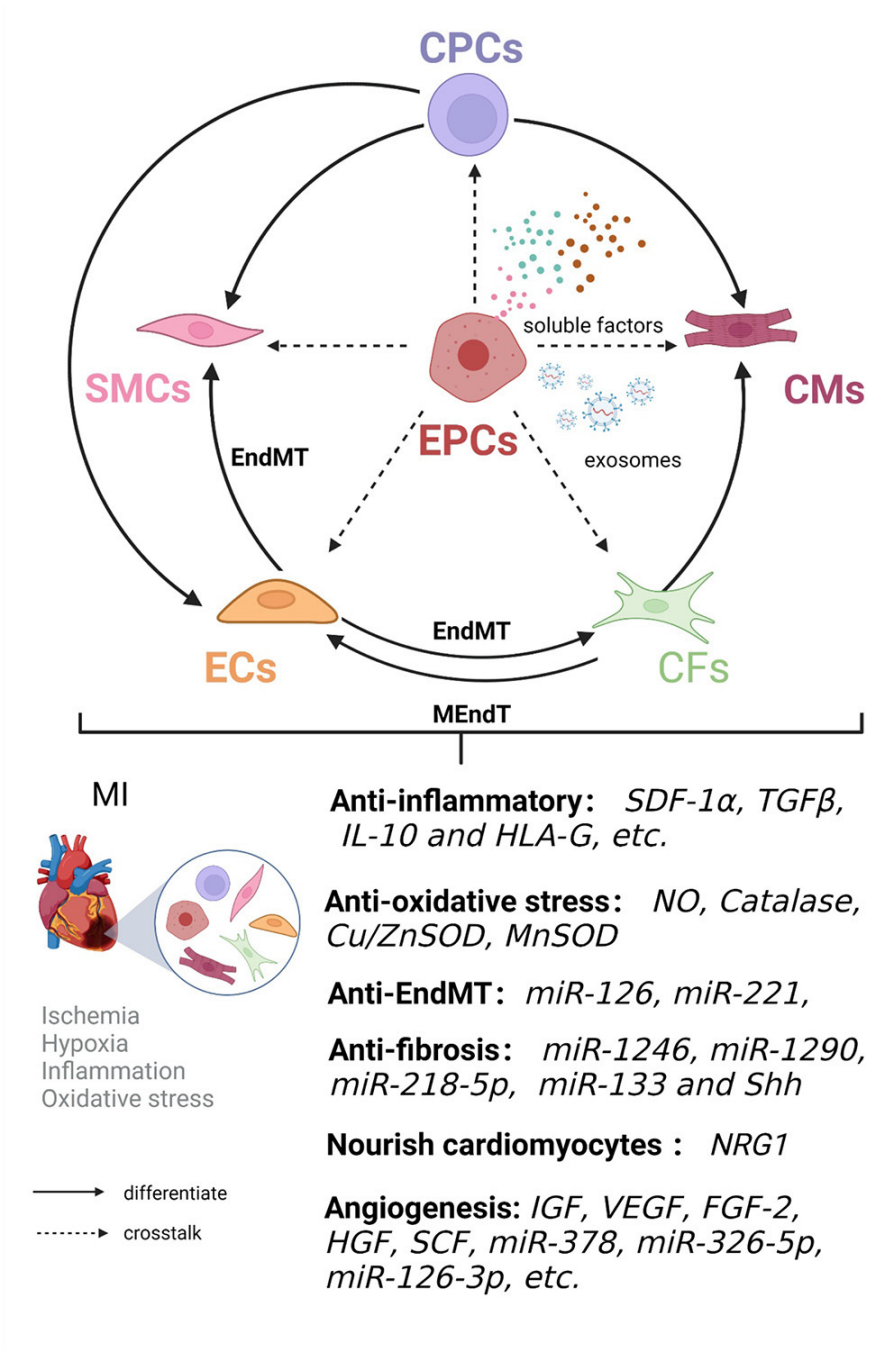
Crosstalk between EPCs and cardiac cell populations is involved in cardiac remodeling and repair in ischemic heart disease. EPCs have the ability to differentiate into endothelial cells in an autocrine and paracrine manner. EPC crosstalk with endothelial cells (ECs), smooth muscle cells (SMCs), cardiomyocytes (CMs), cardiac fibroblasts (CFs), and cardiac progenitor cells (CPCs) can jointly improve the microenvironment in terms of inflammation and oxidative stress and inhibit endothelial-mesenchymal transition (EndMT) and fibrosis, nourish cardiomyocytes and induce angiogenesis, thereby promoting cardiac remodeling and repair.

The adult heart is composed of a heterogeneous cell populations comprising 11 major cell types: atrial CMs, ventricular CMs, CFs, ECs, pericytes, SMCs, immune cells (myeloid and lymphoid cells), adipocytes, mesothelial cells, and neuronal cells (89). EPCs promote cardiac repair through direct cell contact and autocrine, and paracrine effects (90). EPC crosstalk with cardiac cell populations promote angiogenesis, improve cardiac microenvironment homeostasis, alleviate adverse remodeling after infarction, and enhance ventricular function (Figure 2, Table 2).

**Table 2:** EPC interactions with cardiac cell populations and mesenchymal stem cells in ischemic heart disease.

**Targeted cell type**	**EPCs/EPC-EXs**	**Factors**	**Mechanism**	**Function**	**References**
ECs	EPCs	IGF, VEGF, FGF-2, HGF, SCF	-	Reduces apoptosis and promotes angiogenesis in HUVECs	(91)
ECs	EPCs	Cu/ZnSOD, MnSOD, catalase, Bcl-2	-	Enhances HUVECs resistance to ROS toxicity and anti-apoptosis	(26)
ECs	EPCs	miR-126	PIK3R2/PI3K/Akt signaling pathway	Inhibits the EndMT process of TGFβ-induced EPCs	(92)
ECs	EPCs	miR-221	PTEN/FoxO3a signaling pathway	Inhibits the EndMT process of TGF-β-induced EPCs	(93)
ECs	EPCs	miR-378	-	Promotes migration and angiogenesis of HUVECs	(94)
ECs	EPCs	miR-326-5p	Wnt1	Enhances the incorporation of EPCs into tubular structures formed by HUVECs *in vitro*, increases angiogenesis, improves LVEF and reduces myocardial fibrosis *in vivo*	(36)
ECs	EPCs	miR-126-3p	-	Reduces the expression of inflammatory factors and promote angiogenesis	(95)
ECs	EPC-EXs	miR-126	PI3K/eNOS/NO signaling pathway	Attenuates ROS-induced ECs damage and apoptosis	(27)
ECs	IL-10KO-EPC-Exs	ILK	NF-kB signaling pathway	Enhances inflammatory response and inhibit ECs angiogenesis	(96)
ECs	IL-10KO-EPC-Exs	miR-375	PDK-1/AKT signaling pathway	Promotes apoptosis of ECs and attenuates angiogenesis	(97)
SMCs	EPCs	SDF1α, VEGF, HGF, TGFβ	-	Stimulates angiogenesis and cardiomyocyte proliferation	(98)
CMs	EPCs	NRG1	PI3K/Akt signaling pathway	Reduces apoptosis and increases proliferation of human pluripotent stem cell-derived cardiomyocytes	(30)
CFs	EPC-EXs	miR-1246/ miR-1290	ELF5/SP1	Promotes MEndT in CFs, promotes angiogenesis, improves cardiac function and reduces cardiac fibrosis	(99)
CFs	EPC-EXs	miR-218-5p/miR-363-3p	p53/JMY	Promotes MEndT in CFs, promotes angiogenesis, improves cardiac function and reduces cardiac fibrosis	(100)
CFs	EPC-EXs	miR-133	-	Promotes MEndT in CFs, promotes angiogenesis	(28)
CFs	EPC-EXs	Shh	Shh signaling pathway	Mediates the transdifferentiation of resident CFs into ECs	(101)
CPCs	EPCs	VEGF, SDF-1, IGF-1	-	Enhances the migration of mature ECs and tissue-resident CPCs.	(102)
MSCs	EPCs	connexin 43, integrin alpha-5	-	Enhances the survival and therapeutic properties of MSCs	(103)

### Endothelial Cells

ECs form a monolayer on the blood vessel wall and mediate the exchange of molecules between the blood and surrounding tissues and maintain the homeostasis of blood vessels (104). EPCs are precursors of ECs, and BM-EPCs can migrate to sites of ischemic injury, integrate into the original vasculature, differentiate into ECs, and restore the integrity of vascular ECs and their functions (25, 105). On the other hand, EPC-derived anti-apoptotic and pro-angiogenic factors (such as IGF, VEGF, FGF-2, HGF, and SCF) and anti-inflammatory factors stimulate the original ECs and promote new blood vessel formation (91).

EC dysfunction is defined as a state in which reduced NO bioavailability and increased ROS-related oxidative stress cause impaired vasodilation and promote inflammation and coagulation (106). EPCs have been shown to express eNOS, and activated eNOS promotes NO production to prevent EC damage (107). In ischemic or inflammatory tissue microenvironments with elevated ROS levels, the expression of catalase, copper-zinc superoxide dismutase (Cu/ZnSOD), manganese superoxide dismutase (MnSOD) and anti-apoptotic factor Bcl-2 in EPCs was increased, supporting the supposition that ECs are resistant to ROS-induced toxicity, thereby maintaining their viability and functional activity (26). In addition, recent studies have shown that the endothelial-mesenchymal transition (EndMT) is an important process in vascular endothelial injury and is closely related to plaque stability and endothelial microenvironment homeostasis in coronary atherosclerosis (108). The EndMT causes ECs to lose their endothelial characteristics and acquire a mesenchymal-like cell (SMC and fibroblast) phenotype (109, 110). EPCs undergo the EndMT to differentiate into smooth muscle-like cells after treatment with TGFβ, and TGF-β exacerbates promotes cardiac fibrosis by inducing the EndMT (109, 111). Zhang et al. found that overexpression of miR-126 inhibited the EndMT of EPCs, suggesting that intimal hyperplasia in CAD is inhibited through the EndMT (92). Moreover, the upregulation of miR-221 expression repressed the EndMT of EPCs, possibly by interacting with PTEN to regulate FoxO3a signaling, and promoted EPC acquisition of the endothelial phenotype (93).

Numerous studies have confirmed that EPCs and EPC-extracellular vesicle (EV)-carried miRNAs have angiogenic/vasculogenic properties (112). Templin et al. isolated CD34+ cells from patients with acute ST-segment-elevation myocardial infarction (STEMI) and found that miR-378 expression was significantly upregulated and may have promoted angiogenesis through paracrine vascular growth factor signaling (94). Overexpression of miR-326-5p and miR-126-3p in EPCs significantly promoted tubular structure formation and angiogenesis. Furthermore, miR-326-5p- and miR-126-3p-overexpressing EPCs transplanted into an AMI animal model promoted a significant increase in angiogenesis in the area surrounding MI-damaged tissue and improved left ventricular hemodynamic function (36, 95). EPC-MV-carried miR-126 attenuated ROS-induced vascular endothelial injury and EC apoptosis in a hypoxia/reoxygenation (H/R) injury model (27). In addition, the content of EPC-EVs changes under inflammatory conditions and impairs the repair ability of EPC-EVs in ischemic heart tissue. Yue et al. found specific enrichment of integrin-linked kinase (ILK) and miR-375 in IL-10KO-EPC-Exos by IL-10 knockout mimicking systemic inflammatory conditions. By knocking down ILK and miR-375 in IL-10KO EPC-Exos, NF-kB activation was inhibited in endothelial cells, and the inflammation-induced apoptosis and angiogenic capacity of EPCs and ECs were rescued (96, 97).

### Smooth Muscle Cells

Mature vasculature requires ECs and SMCs, while angiogenesis requires dynamically regulated interactions between ECs, SMCs, and angiogenic factors (96). After vascular injury, EPCs regulate the proliferation, migration, secretion capacity, and phenotypic switching of SMCs, promoting SMCs to form neointima (97). Coculturing EPCs with SMCs induced neovascular development of capillary networks and prevascularized structures, enhanced angiogenic responses and induced the formation of mature blood vessels (113). The interaction between EPCs and SMCs stimulated a massive release of SDF1α, VEGF, HGF, and TGFβ, which stimulated CM proliferation and angiogenesis (113). EPC and SMC bilayer cell sheets were transplanted into MI rats, and the interaction of these two cell types in the animal model enhanced arterialization, thereby reducing myocardial fibrosis and adverse remodeling after MI (113, 114). A similar study showed that a bilayer of EPCs and SMCs also attenuated cardiac fibrosis and ventricular remodeling in diabetic cardiomyopathy and improved cardiac function (98).

### Cardiomyocytes

CMs constitute the major cell type lost during ischemia, MI and heart failure; however, CMs show a limited ability to proliferate, and adults exhibit low turnover rates of newly formed CMs, ~0.5–2% per year (115). Current therapeutic goals involve reducing myocardial fibrosis, alleviating myocardial cell dysfunction, and promoting myocardial regeneration. In a myocardial ischemia–reperfusion model, EPCs and EPC-derived conditioned medium enhanced tissue regeneration and improved ischemia-related organ dysfunction by inhibiting oxidative stress (oxidative proteins and markers of oxidative stress), autophagy (LC3B-I and LC3B-II), apoptosis (cleaved caspase 3 and cleaved PARP) and fibrosis (Smad3 and TGF-ß)-associated marker expression (116). Studies have shown that miR-214 expression was upregulated in EPCs and may have regulated CM Ca2+ homeostasis and cell survival during myocardial ischemia injury through miR-214 (117, 118). Hong et al. found that crosstalk between ECs and CMs was critical for regulating CM function and that vascular networks generated by ECFCs enhanced the engraftment of human pluripotent stem cells. The cardioprotective factor NRG1, which is highly expressed in ECFCs, exhibited high paracrine signaling for differentiation of human pluripotent stem cell-derived CMs by activating the PI3K/Akt signaling pathway (30).

### Cardiac Fibroblasts

CFs are essential for cardiac tissue structural remodeling; cardiac chemical, mechanical and electrophysiological properties; and angiogenesis (119). After cardiac injury, CFs transdifferentiate into myofibroblasts and simultaneously trigger the secretion of high levels of extracellular matrix components such as type I collagen, type III collagen, elastin (such as α-smooth muscle actin), fibronectin, and fibrin, leading to the destruction of normal myocardial structure and increased fibrosis (120). Fibroblasts acquire endothelial-like functions through the mesenchymal-endothelial transition (MEndT) and participate in angiogenesis in damaged heart areas, reversing myocardial fibrosis (121).

EXs derived from EPCs induced the upregulated expression of MEndT-related genes and reduced high mobility box 1 protein B1 expression to promote CF differentiation into ECs (122). Reduced basic fibroblast growth factor and increased angiogenesis in ischemic hearts after EPC EX treatment reduced the area of cardiac fibrosis. Therefore, it was inferred that the proangiogenic function of EPC-derived EXs may be partially attributable of the activated MEndT of CFs (117). However, how EPC-derived EXs regulate the MEndT has not been elucidated to date. By performing a microarray analysis, Huang et al. found differential expression of miRNAs in EPCs and EPC-derived EXs carrying miR-1246 and miR-1290 targeted the binding of the transcription factors ELF5 and SP1 in fibroblasts, inducing endothelial marker CD31 expression (123). In a prior study, p53 signaling was found to activate fibroblast MEndT, enhance vascularity, and improve cardiac function (121). Ke et al. found that EPC-EV-derived miRNAs, specifically the upregulated expression of p53 by miR-218-5p and the downregulated expression of JMY by miR-363-3p, might have alleviated myocardial fibrosis and improved cardiac function by inducing the MEndT to increase angiogenesis (124). Lin et al. performed YBX-1-mediated sorting of miR-133 into H/R-induced EPC-derived EXs and found that they increased fibroblast angiogenesis and the MEndT rate (28). Interestingly, EXs containing Shh protein secreted by Shh-modified CD34+ cells may have mediated the transdifferentiation of resident fibroblasts to ECs by activating the Shh signaling pathway (99). Additionally, Cao et al. found that fibroblasts were efficiently transformed into cardiomyocyte-like cells after reprogramming, providing new insights into cardiac regeneration therapy (100).

### Cardiac Progenitor Cells

A population of resident cardiac stem cells, namely, CPCs, has been isolated from percutaneous endomyocardial biopsy specimens and they showed the potential to differentiate into CMs, SMCs, and ECs (101). Partially through the endogenous repair program in the heart and possibly through the activation of endogenous CPCs and CM proliferation, lost CMs are replaced with new cardiomyocytes to promote the recovery of cardiac function (125). Soluble factors released by EPCs promoted the mobilization and recruitment of circulating and tissue-resident progenitor cells into ischemic tissue under ischemic hypoxic pathological conditions and supported tissue-resident cell (such as mature EC or CPCs) survival and function through paracrine signaling (29). Balbi et al. found that the human amniotic fluid stem cell secretome induced the activation of endogenous epicardial progenitor cells and Ca2+-dependent angiogenesis in ECFCs after ischemia-hypoxia injury (126). Deutsch et al. found that ECFC treatment stimulated robust endogenous angiogenesis in Sca-1+ cardiac progenitors, which was accompanied with an increase in the blood vessels formed following infusion of ECFCs into—ischemic myocardium, while an increase in Sca1+ cardiac-resident progenitors was involved in adverse remodeling after MI (127). In another study, engraftment of Tβ4-treated diabetic EPCs significantly increased the capillary density and attracted an increasing number of c-Kit-positive progenitors into the infarcted heart to enhance repair mechanisms (128).

### Crosstalk Between EPCs and MSCs

MSCs are adult stem cells derived from BM and show multidirectional differentiation potential. In the treatment of CVDs, MSCs have the ability to differentiate into CMs and vascular system cells and exert anti-inflammatory, antifibrotic and proangiogenic effects (102, 129). Crosstalk between MSCs and EPCs enhances cardiac repair and cardiac function after MI through paracrine signaling and direct cell contact (130, 131). On the one hand, coculturing MSCs and EPCs enhanced the therapeutic properties of the MSCs, and up-regulation of connexin 43 and integrin-5 expression promoted local intercellular communication and increased MSC engraftment integration capacity (132). Paracrine factors in EPCs stimulated MSCs while maintaining the adhesion and proliferation properties of ECs, thereby supporting efficient angiogenesis (133). On the other hand, coculturing enhanced the angiogenic properties of the EPCs. Both MSCs and EPCs secrete angiogenic factors; however, MSCs secrete additional proangiogenic factors (VEGF and IGFBP-3) that promote the migration, invasion and angiogenesis of EPCs (132).

### The Application and Limitations of EPC Therapy and Clinical Translation

**Figure 3:**
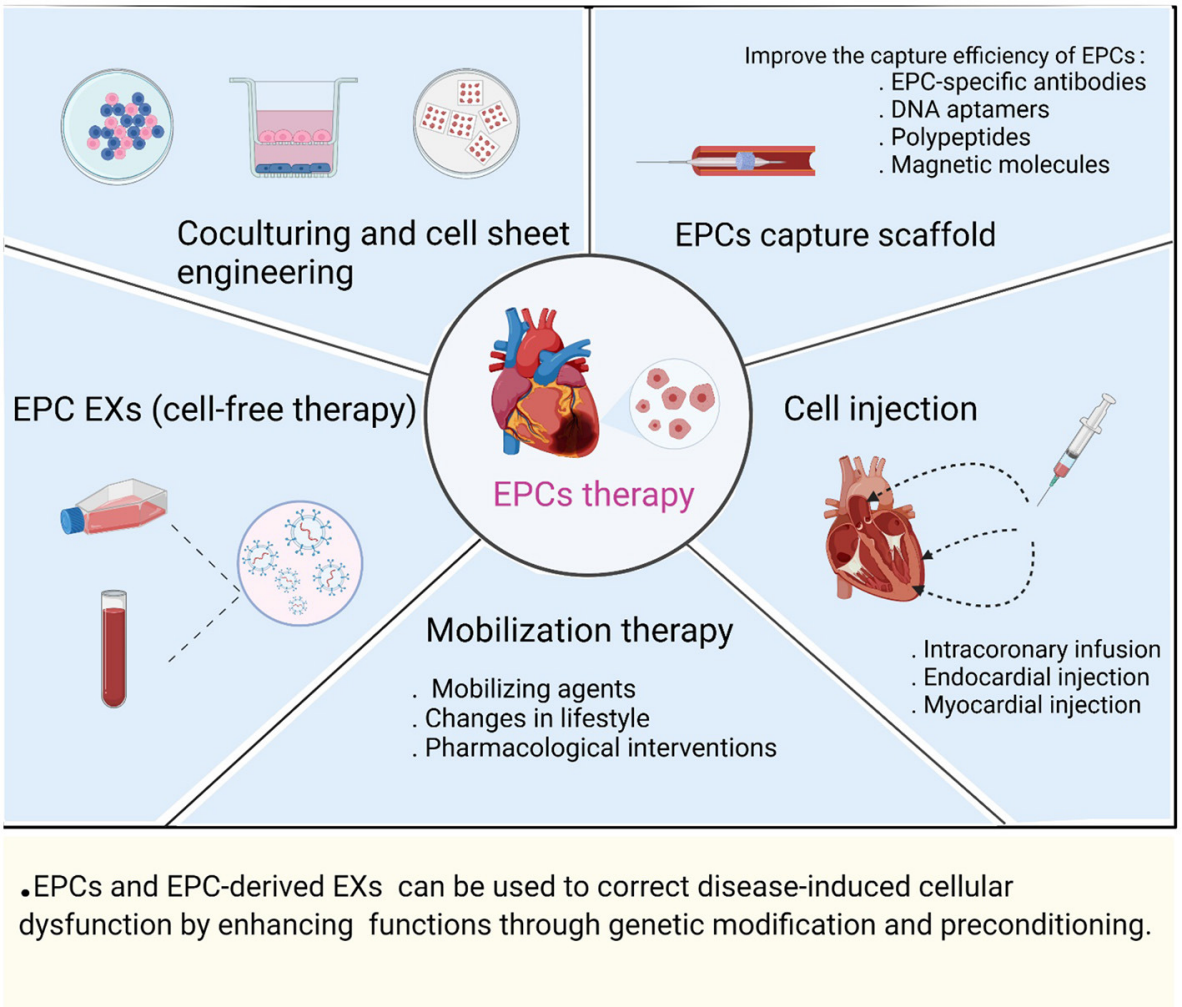
Preclinical and clinical applications of EPC therapy.

Clinical studies have demonstrated that EPCs can be used as biomarkers of CVD progression and risk, while transplanted EPCs exert paracrine-signaling-induced effects on vascular remodeling, angiogenesis, and tissue repair in the treatment of ischemic disease. The main applications of EPC therapy are currently involve: (1) mobilization therapy; (2) EPC capture scaffolds; (3) cell injection; (4) coculturing and cell sheet engineering; and (5) EPC EXs (cell-free therapy) (Figure 3).

EPCs are sources of endogenous repair cells, the number and functional activity of EPCs are regulated by cardiovascular risk factors, and the mobilization of EPCs can be regulated by interventions such as treatment with various mobilizing agents (chemokines, growth factors, and cytokines), changes in lifestyle (appropriate physical activity, diet, and smoking cessation) and pharmacological interventions (8, 103, 134, 135). To ensure successful EPC engraftment and promote rapid endothelialization, circulating EPCs can be captured by an EPCs scaffold; that is, EPCs bind to the scaffold surface, and then, the EPCs differentiate into a functional endothelial layer (136). To date, EPC-specific antibodies (monoclonal antibodies against CD133, CD34, and CD146), aptamers (DNA aptamers), polypeptides (Arg-Gly-Asp peptides), and magnetic molecules (superparamagnetic iron oxide nanoparticles) have been widely used in cardiovascular biomaterials to improve the therapeutic effect of EPC capture scaffolds (137). Multiple research groups have evaluated the safety and feasibility of EPCs via endocardial injection, direct injection into the infarcted myocardium, and intracoronary infusion in CVD (angina, MI, and heart failure) (138–140). Stamm et al. found that injection of purified CD133+ progenitor cells into the myocardial infarct border zone during CABG found that left ventricular ejection fraction (LVEF) was significantly higher in the CABG and cell combination treatment group than in the CABG-only group after 6 months (139). Salvatore et al., studying intracoronary infusion of CD34+ cells in patients with end-stage diffuse CAD who were not candidates for coronary interventional therapy, performed a 5-year follow-up to evaluate the potential of CD34+ cell therapy in improving left ventricular function, heart failure, and cardiac remodeling (140). They found that cell sheet engineering of bioengineered tissues in the repaired heart led to more efficient cell delivery than intracoronary or myocardial injection (141). The implantation of double-layered cell sheets constructed by coculturing EPCs with SMCs or MSCs improved the ability of tissues to form capillary-like networks and a functional vascular bed, while coculturing stimulated mutual paracrine effects to enhance cell survival and differentiation (113, 114, 132). New regenerative medicine approaches were used to treat CVDs through cell-free therapy by exploiting the paracrine signaling mechanisms of stem and progenitor cells, EXs derived from EPCs that carry proangiogenic and cardioprotective cargo, and transplantation of EPC EXs to promote myocardial angiogenesis and recovery of cardiac function after infarction (142). Chen et al. demonstrated sustained delivery of EPC-derived EVs to the ischemic myocardium via injectable hydrogels in a rat model of MI, followed by uptake by ECs, increased vascular density in the infarcted area, and improved cardiac hemodynamics and cardiac reshaping (143). The successful regeneration capacity mediated through cell therapy requires consideration of key factors such as the source of the transplanted cells, dose of the cells, efficacy of the cells, delivery time, and route of administration (144). Currently, EPCs are inconsistently defined, and therefore, the cell types used in clinical trials vary, particularly in terms of cell isolation, culturing methods, and characterization of cell surface markers. In addition, the cell dose and route of administration vary from trial to trial, making it difficult to compare results from different clinical trials (145). Studies have shown that obtaining sufficient ECFCs from PB is difficult in many cases; however, ECFCs isolated from umbilical cord blood show high proliferative potential and contain relatively high levels of telomerase activity to prevent aging (10). Human induced pluripotent stem cells can reproducibly form isolated homogeneous and stable populations with umbilical cord blood ECFC properties, which include high proliferative and no teratoma-forming potential (146). In addition, different degrees of tissue ischemia, hypoxia, inflammation, and oxidative stress in ischemic diseases cause EPCs to exhibit reduced proliferative capacity, dysfunction, and reduced angiogenic capacity, resulting in a low survival rate of transplanted cells in host tissues (147). EPCs and EPC-derived EXs can be used to correct disease-induced cellular dysfunction by enhancing *in vivo* functions through genetic modification and preconditioning. For example, the combination of gene modification realized by overexpressing genes such as the miR-326-5p (36), miR-126-3p (95), sonic hedgehog (99), and IL-10 (77) genes in EPCs and subsequent EPC transplantation into AMI animal models was found to be significantly correlated with EPC function and cardiac function recovery. Treatment with high-dose statins before PCI promoted an increase in EPC abundance (148). Thymosin β4 preconditioning enhanced the survival and angiogenic capacity of EPCs during MI and enhanced the repair of the infarcted myocardium (149).

## Conclusion

Accumulating evidence suggests that EPCs are important players in endothelial dysfunction repair and angiogenesis, offering great promise for the treatment of CVD. On the one hand, EPCs promote angiogenesis and vascular remodeling in ischemic tissue and accelerate the process of IHD repair. On the other hand, the paracrine mechanism of EPCs improves the microenvironment after myocardial infarction, enhances cardiac remodeling and repair, and improves cardiac function. In recent years, great progress has been made in research on the role of EPCs in ischemic heart disease, stroke, pulmonary hypertension, and diabetes. However, there are some pressing issues that need to be addressed in future research and clinical trials. How can EPCs be used to establish clear/uniform criteria for definition, separation and quantification? How do circulating EPCs precisely and specifically target ischemic and damaged tissue? How can EPCs reduce the negative effects of ischemia, hypoxia, inflammation, and oxidative stress in disease settings? The paracrine signaling mechanism of EPCs, which comprises a complex network of crosstalk with other cardiac cell types and stem progenitor cells, serves an important repair function; however, the mechanisms of action of specific paracrine components (cytokines, proteins, and miRNAs), the specific cells targeted by EPCs, and the communication and signal transduction mechanisms between EPCs and other types of cells have not been fully elucidated. Therefore, further studies on the molecular mechanisms of these biological processes are expected to contribute to the translation of EPC therapy to the practice of precision medicine.

## Author Contributions

HH wrote the manuscript and figure legends and created the figures and tables. WH revised the manuscript. All authors contributed to the article and approved the submitted version.

## Funding

The present study was funded by the National Natural Science Foundation of China (Nos. 8176090090 and 81960056), the Guangxi Natural Science Foundation (No. 2017GXNSFAA198147) and the Promotional Project of Guangxi Medical and Health Appropriate Technology (No. S201518).

## Conflict of Interest

The authors declare that the research was conducted in the absence of any commercial or financial relationships that could be construed as a potential conflict of interest.

## Publisher's Note

All claims expressed in this article are solely those of the authors and do not necessarily represent those of their affiliated organizations, or those of the publisher, the editors and the reviewers. Any product that may be evaluated in this article, or claim that may be made by its manufacturer, is not guaranteed or endorsed by the publisher.

## References

[1] Roth GA Mensah GA Johnson CO Addolorato G Ammirati E Baddour LM. Global burden of cardiovascular diseases and risk factors, 1990-2019: update from the GBD 2019 Study. J Am Coll Cardiol. (2020) 76:2982–3021. 10.1016/j.jacc.2020.11.010 33309175 PMC7755038

[2] Prabhu SD Frangogiannis NG. The biological basis for cardiac repair after myocardial infarction: from inflammation to fibrosis. Circ Res. (2016) 119:91–112. 10.1161/CIRCRESAHA.116.303577 27340270 PMC4922528

[3] Cohn JN Ferrari R Sharpe N. Cardiac remodeling–concepts and clinical implications: a consensus paper from an international forum on cardiac remodeling. Behalf of an International Forum on Cardiac Remodeling. J Am Coll Cardiol. (2000) 35:569–82. 10.1016/S0735-1097(99)00630-0 10716457

[4] Segers VFM De Keulenaer GW. Autocrine signaling in cardiac remodeling: a rich source of therapeutic targets. J Am Heart Assoc. (2021) 10:e019169. 10.1161/JAHA.120.019169 33470124 PMC7955414

[5] Serruys PW Morice MC Kappetein AP Colombo A Holmes DR Mack MJ. Percutaneous coronary intervention versus coronary-artery bypass grafting for severe coronary artery disease. N Engl J Med. (2009) 360:961–72. 10.1056/NEJMoa0804626 19228612

[6] Arjmand B Abedi M Arabi M Alavi-Moghadam S Rezaei-Tavirani M Hadavandkhani M. Regenerative medicine for the treatment of ischemic heart disease; status and future perspectives. Front Cell Dev Biol. (2021) 9:704903. 10.3389/fcell.2021.704903 34568321 PMC8461329

[7] Khakoo AY Finkel T. Endothelial progenitor cells. Annu Rev Med. (2005) 56:79–101. 10.1146/annurev.med.56.090203.104149 15660503

[8] Hill JM Zalos G Halcox JP Schenke WH Waclawiw MA Quyyumi AA. Circulating endothelial progenitor cells, vascular function, and cardiovascular risk. N Engl J Med. (2003) 348:593–600. 10.1056/NEJMoa022287 12584367

[9] Asahara T Murohara T Sullivan A Silver M van der Zee R Li T. Isolation of putative progenitor endothelial cells for angiogenesis. Science. (1997) 275:964–7. 10.1126/science.275.5302.964 9020076

[10] Ingram DA Mead LE Tanaka H Meade V Fenoglio A Mortell K. Identification of a novel hierarchy of endothelial progenitor cells using human peripheral and umbilical cord blood. Blood. (2004) 104:2752–60. 10.1182/blood-2004-04-1396 15226175

[11] Gremmels H Fledderus JO van Balkom BW Verhaar MC. Transcriptome analysis in endothelial progenitor cell biology. Antioxid Redox Signal. (2011) 15:1029–42. 10.1089/ars.2010.3594 20812873

[12] Medina RJ Barber CL Sabatier F Dignat-George F Melero-Martin JM Khosrotehrani K. Endothelial progenitors: a consensus statement on nomenclature. Stem Cells Transl Med. (2017) 6:1316–20. 10.1002/sctm.16-0360 28296182 PMC5442722

[13] Patel J Donovan P Khosrotehrani K. Concise review: functional definition of endothelial progenitor cells: a molecular perspective. Stem Cells Transl Med. (2016) 5:1302–6. 10.5966/sctm.2016-0066 27381992 PMC5031185

[14] Dight J Zhao J Styke C Khosrotehrani K Patel J. Resident vascular endothelial progenitor definition and function: the age of reckoning. Angiogenesis. (2021) 25:15–33. 10.1007/s10456-021-09817-2 34499264 PMC8813834

[15] Peichev M Naiyer AJ Pereira D Zhu Z Lane WJ Williams M. Expression of VEGFR-2 and AC133 by circulating human CD34(+) cells identifies a population of functional endothelial precursors. Blood. (2000) 95:952–8. 10.1182/blood.V95.3.952.003k27_952_958 10648408

[16] Guerin CL Guyonnet L Goudot G Revets D Konstantinou M Chipont A. Multidimensional proteomic approach of endothelial progenitors demonstrate expression of KDR restricted to CD19 cells. Stem Cell Rev Rep. (2021) 17:639–51. 10.1007/s12015-020-10062-1 33205351 PMC7670993

[17] Steffen E Mayer von Wittgenstein WBE Hennig M Niepmann ST Zietzer A Werner N. Murine sca1/flk1-positive cells are not endothelial progenitor cells, but B2 lymphocytes. Basic Res Cardiol. (2020) 115:18. 10.1007/s00395-020-0774-6 31980946 PMC6981106

[18] Fujisawa T Tura-Ceide O Hunter A Mitchell A Vesey A Medine C. Endothelial progenitor cells do not originate from the bone marrow. Circulation. (2019) 140:1524–6. 10.1161/CIRCULATIONAHA.119.042351 31657952 PMC6818974

[19] Ingram DA Mead LE Moore DB Woodard W Fenoglio A Yoder MC. Vessel wall-derived endothelial cells rapidly proliferate because they contain a complete hierarchy of endothelial progenitor cells. Blood. (2005) 105:2783–6. 10.1182/blood-2004-08-3057 15585655

[20] Chambers SEJ Pathak V Pedrini E Soret L Gendron N Guerin CL. Current concepts on endothelial stem cells definition, location, and markers. Stem Cells Transl Med. (2021) 10:S54–S61. 10.1002/sctm.21-0022 34724714 PMC8560200

[21] Abdelgawad ME Desterke C Uzan G Naserian S. Single-cell transcriptomic profiling and characterization of endothelial progenitor cells: new approach for finding novel markers. Stem Cell Res Ther. (2021) 12:145. 10.1186/s13287-021-02185-0 33627177 PMC7905656

[22] Zhu Y Yang T Duan J Mu N Zhang T. MALAT1/miR-15b-5p/MAPK1 mediates endothelial progenitor cells autophagy and affects coronary atherosclerotic heart disease via mTOR signaling pathway. Aging. (2019) 11:1089–109. 10.18632/aging.101766 30787203 PMC6402525

[23] Schuh A Liehn EA Sasse A Hristov M Sobota R Kelm M. Transplantation of endothelial progenitor cells improves neovascularization and left ventricular function after myocardial infarction in a rat model. Basic Res Cardiol. (2008) 103:69–77. 10.1007/s00395-007-0685-9 17999028

[24] Cheng Y Guo S Liu G Feng Y Yan B Yu J. Transplantation of bone marrow-derived endothelial progenitor cells attenuates myocardial interstitial fibrosis and cardiac dysfunction in streptozotocin-induced diabetic rats. Int J Mol Med. (2012) 30:870–6. 10.3892/ijmm.2012.1083 22859217

[25] Jujo K Ii M Losordo DW. Endothelial progenitor cells in neovascularization of infarcted myocardium. J Mol Cell Cardiol. (2008) 45:530–44. 10.1016/j.yjmcc.2008.08.003 18755197 PMC2628572

[26] Yang Z von Ballmoos MW Faessler D Voelzmann J Ortmann J Diehm N. Paracrine factors secreted by endothelial progenitor cells prevent oxidative stress-induced apoptosis of mature endothelial cells. Atherosclerosis. (2010) 211:103–9. 10.1016/j.atherosclerosis.2010.02.022 20227693

[27] Wang J Chen S Ma X Cheng C Xiao X Chen J. Effects of endothelial progenitor cell-derived microvesicles on hypoxia/reoxygenation-induced endothelial dysfunction and apoptosis. Oxid Med Cell Longev. (2013) 2013:572729. 10.1155/2013/572729 24288585 PMC3830832

[28] Lin F Zeng Z Song Y Li L Wu Z Zhang X. YBX-1 mediated sorting of miR-133 into hypoxia/reoxygenation-induced EPC-derived exosomes to increase fibroblast angiogenesis and MEndoT. Stem Cell Res Ther. (2019) 10:263. 10.1186/s13287-019-1377-8 31443679 PMC6708233

[29] Urbich C Aicher A Heeschen C Dernbach E Hofmann WK Zeiher AM. Soluble factors released by endothelial progenitor cells promote migration of endothelial cells and cardiac resident progenitor cells. J Mol Cell Cardiol. (2005) 39:733–42. 10.1016/j.yjmcc.2005.07.003 16199052

[30] Hong X Oh N Wang K Neumeyer J Lee CN Lin RZ. Human endothelial colony-forming cells provide trophic support for pluripotent stem cell-derived cardiomyocytes via distinctively high expression of neuregulin-1. Angiogenesis. (2021) 24:327–44. 10.1007/s10456-020-09765-3 33454888 PMC8337094

[31] de la Puente P Muz B Azab F Azab AK. Cell trafficking of endothelial progenitor cells in tumor progression. Clin Cancer Res. (2013) 19:3360–8. 10.1158/1078-0432.CCR-13-0462 23665736

[32] Chavakis E Dimmeler S. Homing of progenitor cells to ischemic tissues. Antioxid Redox Signal. (2011) 15:967–80. 10.1089/ars.2010.3582 20812875

[33] Busch A Eken SM Maegdefessel L. Prospective and therapeutic screening value of non-coding RNA as biomarkers in cardiovascular disease. Ann Transl Med. (2016) 4:236. 10.21037/atm.2016.06.06 27429962 PMC4930509

[34] Guduric-Fuchs J Pedrini E Lechner J Chambers SEJ O'Neill CL Mendes Lopes de Melo J. miR-130a activates the VEGFR2/STAT3/HIF1α axis to potentiate the vasoregenerative capacity of endothelial colony-forming cells in hypoxia. Mol Ther Nucleic Acids. (2021) 23:968–81. 10.1016/j.omtn.2021.01.015 33614244 PMC7869000

[35] Lu WJ Liang HB Li YF Tu XQ He JR Ding KQ. MicroRNA-210-3p targets RGMA to enhance the angiogenic functions of endothelial progenitor cells under hypoxic conditions. Front Cell Neurosci. (2019) 13:223. 10.3389/fncel.2019.00223 31164807 PMC6536652

[36] Li X Xue X Sun Y Chen L Zhao T Yang W. MicroRNA-326-5p enhances therapeutic potential of endothelial progenitor cells for myocardial infarction. Stem Cell Res Ther. (2019) 10:323. 10.1186/s13287-019-1413-8 31730013 PMC6858781

[37] Kong Z Wang Y Zhang Y Shan W Wu J Wang Q. MicroRNA-126 promotes endothelial progenitor cell proliferation and migration ability via the Notch pathway. Cardiovasc Diagn Ther. (2020) 10:490–9. 10.21037/cdt-20-178 32695628 PMC7369286

[38] Wu Q Qi B Duan X Ming X Yan F He Y. MicroRNA-126 enhances the biological function of endothelial progenitor cells under oxidative stress via PI3K/Akt/GSK3β and ERK1/2 signaling pathways. Bosn J Basic Med Sci. (2021) 21:71–80. 10.17305/bjbms.2019.4493 31999938 PMC7861621

[39] Chen P Zhong J Ye J He Y Liang Z Cheng Y. miR-324-5p protects against oxidative stress-induced endothelial progenitor cell injury by targeting Mtfr1. J Cell Physiol. (2019) 234:22082–92. 10.1002/jcp.28771 31066044

[40] Liu Y Xu J Gu R Li Z Wang K Qi Y. Circulating exosomal miR-144-3p inhibits the mobilization of endothelial progenitor cells post myocardial infarction via regulating the MMP9 pathway. Aging. (2020) 12:16294–303. 10.18632/aging.103651 32843584 PMC7485705

[41] Li WD Zhou DM Sun LL Xiao L Liu Z Zhou M. LncRNA WTAPP1 promotes migration and angiogenesis of endothelial progenitor cells via MMP1 through MicroRNA 3120 and Akt/PI3K/Autophagy pathways. Stem Cells. (2018) 36:1863–74. 10.1002/stem.2904 30171660

[42] Li C Lin L Zhang L Xu R Chen X Ji J. Long noncoding RNA p21 enhances autophagy to alleviate endothelial progenitor cells damage and promote endothelial repair in hypertension through SESN2/AMPK/TSC2 pathway. Pharmacol Res. (2021) 173:105920. 10.1016/j.phrs.2021.105920 34601081

[43] Yu G Li S Liu P Shi Y Liu Y Yang Z. LncRNA TUG1 functions as a ceRNA for miR-6321 to promote endothelial progenitor cell migration and differentiation. Exp Cell Res. (2020) 388:111839. 10.1016/j.yexcr.2020.111839 31935381

[44] Li Y Zhi K Han S Li X Li M Lian W. TUG1 enhances high glucose-impaired endothelial progenitor cell function via miR-29c-3p/PDGF-BB/Wnt signaling. Stem Cell Res Ther. (2020) 11:441. 10.1186/s13287-020-01958-3 33059750 PMC7558752

[45] Xiao J Lu Y Yang X. THRIL mediates endothelial progenitor cells autophagy via AKT pathway and FUS. Mol Med. (2020) 26:86. 10.1186/s10020-020-00201-2 32907536 PMC7488174

[46] Shi R Jin Y Hu W Lian W Cao C Han S. Exosomes derived from mmu_circ_0000250-modified adipose-derived mesenchymal stem cells promote wound healing in diabetic mice by inducing miR-128-3p/SIRT1-mediated autophagy. Am J Physiol Cell Physiol. (2020) 318:C848–C56. 10.1152/ajpcell.00041.2020 32159361

[47] Cheng J Hu W Zheng F Wu Y Li M. hsa_circ_0058092 protects against hyperglycemia-induced endothelial progenitor cell damage via miR-217/FOXO3. Int J Mol Med. (2020) 46:1146–54. 10.3892/ijmm.2020.4664 32705235 PMC7387092

[48] Tian D Xiang Y Tang Y Ge Z Li Q Zhang Y. Circ-ADAM9 targeting PTEN and ATG7 promotes autophagy and apoptosis of diabetic endothelial progenitor cells by sponging mir-20a-5p. Cell Death Dis. (2020) 11:526. 10.1038/s41419-020-02745-x 32661238 PMC7359341

[49] Bartel DP. MicroRNAs: target recognition and regulatory functions. Cell. (2009) 136:215–33. 10.1016/j.cell.2009.01.002 19167326 PMC3794896

[50] Chang TY Huang TS Wang HW Chang SJ Lo HH Chiu YL. miRNome traits analysis on endothelial lineage cells discloses biomarker potential circulating microRNAs which affect progenitor activities. BMC Genomics. (2014) 15:802. 10.1186/1471-2164-15-802 25236949 PMC4176563

[51] Qu K Wang Z Lin XL Zhang K He XL Zhang H. MicroRNAs: key regulators of endothelial progenitor cell functions. Clin Chim Acta. (2015) 448:65–73. 10.1016/j.cca.2015.06.017 26116893

[52] Li X Wu Z Fu X Han W. Long noncoding RNAs: insights from biological features and functions to diseases. Med Res Rev. (2013) 33:517–53. 10.1002/med.21254 22318902

[53] Memczak S Jens M Elefsinioti A Torti F Krueger J Rybak A. Circular RNAs are a large class of animal RNAs with regulatory potency. Nature. (2013) 495:333–8. 10.1038/nature11928 23446348

[54] Han B Chao J Yao H. Circular RNA and its mechanisms in disease: from the bench to the clinic. Pharmacol Ther. (2018) 187:31–44. 10.1016/j.pharmthera.2018.01.010 29406246

[55] Abbott JD Huang Y Liu D Hickey R Krause DS Giordano FJ. Stromal cell-derived factor-1alpha plays a critical role in stem cell recruitment to the heart after myocardial infarction but is not sufficient to induce homing in the absence of injury. Circulation. (2004) 110:3300–5. 10.1161/01.CIR.0000147780.30124.CF 15533866

[56] Dai X Tan Y Cai S Xiong X Wang L Ye Q. The role of CXCR7 on the adhesion, proliferation and angiogenesis of endothelial progenitor cells. J Cell Mol Med. (2011) 15:1299–309. 10.1111/j.1582-4934.2011.01301.x 21418513 PMC4373330

[57] Huang H Xu Z Qi Y Zhang W Zhang C Jiang M. Exosomes from SIRT1-Overexpressing ADSCs restore cardiac function by improving angiogenic function of EPCs. Mol Ther Nucleic Acids. (2020) 21:737–50. 10.1016/j.omtn.2020.07.007 32771925 PMC7412761

[58] Qiu R Cai A Dong Y Zhou Y Yu D Huang Y. SDF-1α upregulation by atorvastatin in rats with acute myocardial infarction via nitric oxide production confers anti-inflammatory and anti-apoptotic effects. J Biomed Sci. (2012) 19:99. 10.1186/1423-0127-19-99 23170833 PMC3533954

[59] Fan X He L Dai Q He J Chen X Dai X. Interleukin-1beta augments the angiogenesis of endothelial progenitor cells in an NF-kappaB/CXCR7-dependent manner. J Cell Mol Med. (2020) 24:5605–14. 10.1111/jcmm.15220 32239650 PMC7214148

[60] Zheng H Dai T Zhou B Zhu J Huang H Wang M. SDF-1alpha/CXCR4 decreases endothelial progenitor cells apoptosis under serum deprivation by PI3K/Akt/eNOS pathway. Atherosclerosis. (2008) 201:36–42. 10.1016/j.atherosclerosis.2008.02.011 18384792

[61] Shao H Tan Y Eton D Yang Z Uberti MG Li S. Statin and stromal cell-derived factor-1 additively promote angiogenesis by enhancement of progenitor cells incorporation into new vessels. Stem Cells. (2008) 26:1376–84. 10.1634/stemcells.2007-0785 18308946

[62] Tang Y Zhang Y Chen Y Xiang Y Xie Y. Role of the microRNA, miR-206, and its target PIK3C2α in endothelial progenitor cell function – potential link with coronary artery disease. Febs J. (2015) 282:3758–72. 10.1111/febs.13372 26175229

[63] Aicher A Heeschen C Mildner-Rihm C Urbich C Ihling C Technau-Ihling K. Essential role of endothelial nitric oxide synthase for mobilization of stem and progenitor cells. Nat Med. (2003) 9:1370–6. 10.1038/nm948 14556003

[64] Zhou H Tu Q Zhang Y Xie HQ Shuai QY Huang XC. Shear stress improves the endothelial progenitor cell function via the CXCR7/ERK pathway axis in the coronary artery disease cases. BMC Cardiovasc Disord. (2020) 20:403. 10.1186/s12872-020-01681-0 32894067 PMC7487552

[65] Cun Y Diao B Zhang Z Wang G Yu J Ma L. Role of the stromal cell derived factor-1 in the biological functions of endothelial progenitor cells and its underlying mechanisms. Exp Ther Med. (2021) 21:39. 10.3892/etm.2020.9471 33273969 PMC7706408

[66] Hanjaya-Putra D Yee J Ceci D Truitt R Yee D Gerecht S. Vascular endothelial growth factor and substrate mechanics regulate *in vitro* tubulogenesis of endothelial progenitor cells. J Cell Mol Med. (2010) 14:2436–47. 10.1111/j.1582-4934.2009.00981.x 19968735 PMC3823161

[67] Young PP Hofling AA Sands MS. VEGF increases engraftment of bone marrow-derived endothelial progenitor cells (EPCs) into vasculature of newborn murine recipients. Proc Natl Acad Sci USA. (2002) 99:11951–6. 10.1073/pnas.182215799 12195016 PMC129375

[68] Hoffmann BR Wagner JR Prisco AR Janiak A Greene AS. Vascular endothelial growth factor-A signaling in bone marrow-derived endothelial progenitor cells exposed to hypoxic stress. Physiol Genomics. (2013) 45:1021–34. 10.1152/physiolgenomics.00070.2013 24022223 PMC3841787

[69] Everaert BR Van Craenenbroeck EM Hoymans VY Haine SE Van Nassauw L Conraads VM. Current perspective of pathophysiological and interventional effects on endothelial progenitor cell biology: focus on PI3K/AKT/eNOS pathway. Int J Cardiol. (2010) 144:350–66. 10.1016/j.ijcard.2010.04.018 20444511

[70] Chen L Zheng Q Liu Y Li L Chen X Wang L. Adipose-derived stem cells promote diabetic wound healing via the recruitment and differentiation of endothelial progenitor cells into endothelial cells mediated by the VEGF-PLCγ-ERK pathway. Arch Biochem Biophys. (2020) 692:108531. 10.1016/j.abb.2020.108531 32745464

[71] Pei CZ Liu B Li YT Fang L Zhang Y Li YG. MicroRNA-126 protects against vascular injury by promoting homing and maintaining stemness of late outgrowth endothelial progenitor cells. Stem Cell Res Ther. (2020) 11:28. 10.1186/s13287-020-1554-9 31964421 PMC6975061

[72] Ouyang S Li Y Wu X Wang Y Liu F Zhang J. GPR4 signaling is essential for the promotion of acid-mediated angiogenic capacity of endothelial progenitor cells by activating STAT3/VEGFA pathway in patients with coronary artery disease. Stem Cell Res Ther. (2021) 12:149. 10.1186/s13287-021-02221-z 33632325 PMC7905863

[73] Zhang Y Zhao J Lau WB Jiao LY Liu B Yuan Y. Tumor necrosis factor-α and lymphotoxin-α mediate myocardial ischemic injury via TNF receptor 1, but are cardioprotective when activating TNF receptor 2. PLoS ONE. (2013) 8:e60227. 10.1371/journal.pone.0060227 23704873 PMC3660398

[74] Prisco AR Hoffmann BR Kaczorowski CC McDermott-Roe C Stodola TJ Exner EC. Tumor necrosis factor alpha regulates endothelial progenitor cell migration via CADM1 and NF-kB. Stem Cells. (2016) 34:1922–33. 10.1002/stem.2339 26867147 PMC4931961

[75] Goukassian DA Qin G Dolan C Murayama T Silver M Curry C. Tumor necrosis factor-alpha receptor p75 is required in ischemia-induced neovascularization. Circulation. (2007) 115:752–62. 10.1161/CIRCULATIONAHA.106.647255 17261656

[76] Naserian S Abdelgawad ME Afshar Bakshloo M Ha G Arouche N Cohen JL. The TNF/TNFR2 signaling pathway is a key regulatory factor in endothelial progenitor cell immunosuppressive effect. Cell Commun Signal. (2020) 18:94. 10.1186/s12964-020-00564-3 32546175 PMC7298859

[77] Wang Y Chen Q Zhang Z Jiang F Meng X Yan H. Interleukin-10 overexpression improves the function of endothelial progenitor cells stimulated with TNF-α through the activation of the STAT3 signaling pathway. Int J Mol Med. (2015) 35:471–7. 10.3892/ijmm.2014.2034 25504316

[78] Salybekov AA Salybekova AK Pola R Asahara T. Sonic hedgehog signaling pathway in endothelial progenitor cell biology for vascular medicine. Int J Mol Sci. (2018) 19:3040. 10.3390/ijms19103040 30301174 PMC6213474

[79] Fu JR Liu WL Zhou JF Sun HY Xu HZ Luo L. Sonic hedgehog protein promotes bone marrow-derived endothelial progenitor cell proliferation, migration and VEGF production via PI 3-kinase/Akt signaling pathways. Acta Pharmacol Sin. (2006) 27:685–93. 10.1111/j.1745-7254.2006.00335.x 16723086

[80] Lee BNR Son YS Lee D Choi YJ Kwon SM Chang HK. Hedgehog-Interacting Protein (HIP) regulates apoptosis evasion and angiogenic function of late endothelial progenitor cells. Sci Rep. (2017) 7:12449. 10.1038/s41598-017-12571-5 28963460 PMC5622095

[81] Xiao Q Zhao XY Jiang RC Chen XH Zhu X Chen KF. Increased expression of Sonic hedgehog restores diabetic endothelial progenitor cells and improves cardiac repair after acute myocardial infarction in diabetic mice. Int J Mol Med. (2019) 44:1091–105. 10.3892/ijmm.2019.4277 31524224 PMC6657988

[82] Bueno-Beti C Novella S Soleti R Mompeon A Vergori L Sanchis J. Microparticles harbouring Sonic hedgehog morphogen improve the vasculogenesis capacity of endothelial progenitor cells derived from myocardial infarction patients. Cardiovasc Res. (2019) 115:409–18. 10.1093/cvr/cvy189 30124781

[83] Kwon SM Alev C Asahara T. The role of notch signaling in endothelial progenitor cell biology. Trends Cardiovasc Med. (2009) 19:170–3. 10.1016/j.tcm.2009.10.002 20005477

[84] Rizzo P Miele L Ferrari R. The Notch pathway: a crossroad between the life and death of the endothelium. Eur Heart J. (2013) 34:2504–9. 10.1093/eurheartj/ehs141 22645188

[85] Ishige-Wada M Kwon SM Eguchi M Hozumi K Iwaguro H Matsumoto T. Jagged-1 signaling in the bone marrow microenvironment promotes endothelial progenitor cell expansion and commitment of CD133+ human cord blood cells for postnatal vasculogenesis. PLoS ONE. (2016) 11:e0166660. 10.1371/journal.pone.0166660 27846321 PMC5112804

[86] Maeng YS Choi YJ Kim EK. TGFBIp regulates differentiation of EPC (CD133(+) C-kit(+) Lin(-) cells) to EC through activation of the Notch signaling pathway. Stem Cells. (2015) 33:2052–62. 10.1002/stem.2003 25786978

[87] Guo P Li H Chen L Wang D-P Luo Y Xu J. Genetically modified endothelial progenitor cells with hNotch1. ICN overexpression display facilitated angiogenesis. Ann Transl Med. (2020) 8:1316. 10.21037/atm-20-6362 33209896 PMC7661891

[88] Li Y Wang Z Mao M Zhao M Xiao X Sun W. Velvet antler mobilizes endothelial progenitor cells to promote angiogenesis and repair vascular endothelial injury in rats following myocardial infarction. Front Physiol. (2018) 9:1940. 10.3389/fphys.2018.01940 30705637 PMC6344410

[89] Litvinukova M Talavera-Lopez C Maatz H Reichart D Worth CL Lindberg EL. Cells of the adult human heart. Nature. (2020) 588:466–72. 10.1038/s41586-020-2797-4 32971526 PMC7681775

[90] Fadini GP Losordo D Dimmeler S. Critical reevaluation of endothelial progenitor cell phenotypes for therapeutic and diagnostic use. Circ Res. (2012) 110:624–37. 10.1161/CIRCRESAHA.111.243386 22343557 PMC3382070

[91] Ratajczak J Kucia M Mierzejewska K Marlicz W Pietrzkowski Z Wojakowski W. Paracrine proangiopoietic effects of human umbilical cord blood-derived purified CD133+ cells–implications for stem cell therapies in regenerative medicine. Stem Cells Dev. (2013) 22:422–30. 10.1089/scd.2012.0268 23003001 PMC3549621

[92] Zhang J Zhang Z Zhang DY Zhu J Zhang T Wang C. microRNA 126 inhibits the transition of endothelial progenitor cells to mesenchymal cells via the PIK3R2-PI3K/Akt signalling pathway. PLoS ONE. (2013) 8:e83294. 10.1371/journal.pone.0083294 24349482 PMC3862723

[93] Zhou E Zou Y Mao C Li D Wang C Zhang Z. MicroRNA-221 inhibits the transition of endothelial progenitor cells to mesenchymal cells via the PTEN/FoxO3a signaling pathway. Adv Clin Exp Med. (2021) 30:1263–70. 10.17219/acem/141446 34610220

[94] Templin C Volkmann J Emmert MY Mocharla P Muller M Kraenkel N. Increased proangiogenic activity of mobilized CD34+ progenitor cells of patients with acute ST-Segment-Elevation myocardial infarction: role of differential MicroRNA-378 expression. Arterioscler Thromb Vasc Biol. (2017) 37:341–9. 10.1161/ATVBAHA.116.308695 28062497

[95] Li H Liu Q Wang N Xu Y Kang L Ren Y. Transplantation of endothelial progenitor cells overexpressing miR-126-3p improves heart function in ischemic cardiomyopathy. Circ J. (2018) 82:2332–41. 10.1253/circj.CJ-17-1251 29998929

[96] Carmeliet P. Mechanisms of angiogenesis and arteriogenesis. Nat Med. (2000) 6:389–95. 10.1038/74651 10742145

[97] Mause SF Ritzel E Deck A Vogt F Liehn EA. Endothelial progenitor cells modulate the phenotype of smooth muscle cells and increase their neointimal accumulation following vascular injury. Thromb Haemost. (2021) 42:ehab724.3407. 10.1093/eurheartj/ehab724.3407 34214997

[98] Kawamura M Paulsen MJ Goldstone AB Shudo Y Wang H Steele AN. Tissue-engineered smooth muscle cell and endothelial progenitor cell bi-level cell sheets prevent progression of cardiac dysfunction, microvascular dysfunction, and interstitial fibrosis in a rodent model of type 1 diabetes-induced cardiomyopathy. Cardiovasc Diabetol. (2017) 16:142. 10.1186/s12933-017-0625-4 29096622 PMC5668999

[99] Mackie AR Klyachko E Thorne T Schultz KM Millay M Ito A. Sonic hedgehog-modified human CD34+ cells preserve cardiac function after acute myocardial infarction. Circ Res. (2012) 111:312–21. 10.1161/CIRCRESAHA.112.266015 22581926 PMC3511820

[100] Cao N Huang Y Zheng J Spencer CI Zhang Y Fu JD. Conversion of human fibroblasts into functional cardiomyocytes by small molecules. Science. (2016) 352:1216–20. 10.1126/science.aaf1502 27127239

[101] Smith RR Barile L Cho HC Leppo MK Hare JM Messina E. Regenerative potential of cardiosphere-derived cells expanded from percutaneous endomyocardial biopsy specimens. Circulation. (2007) 115:896–908. 10.1161/CIRCULATIONAHA.106.655209 17283259

[102] Premer C Blum A Bellio MA Schulman IH Hurwitz BE Parker M. Allogeneic mesenchymal stem cells restore endothelial function in heart failure by stimulating endothelial progenitor cells. EBioMedicine. (2015) 2:467–75. 10.1016/j.ebiom.2015.03.020 26137590 PMC4485912

[103] Tilling L Chowienczyk P Clapp B. Progenitors in motion: mechanisms of mobilization of endothelial progenitor cells. Br J Clin Pharmacol. (2009) 68:484–92. 10.1111/j.1365-2125.2009.03486.x 19843051 PMC2780273

[104] Pober JS Sessa WC. Evolving functions of endothelial cells in inflammation. Nat Rev Immunol. (2007) 7:803–15. 10.1038/nri2171 17893694

[105] Hu CH Li ZM Du ZM Zhang AX Yang DY Wu GF. Human umbilical cord-derived endothelial progenitor cells promote growth cytokines-mediated neorevascularization in rat myocardial infarction. Chin Med J. (2009) 122:548–55. 10.3760/cma.j.issn.0366-6999.2009.05.012 19323906

[106] Scioli MG Storti G D'Amico F Rodríguez Guzmán R Centofanti F Doldo E. Oxidative stress and new pathogenetic mechanisms in endothelial dysfunction: potential diagnostic biomarkers and therapeutic targets. J Clin Med. (2020) 9:1995. 10.3390/jcm9061995 32630452 PMC7355625

[107] Fleissner F Thum T. Critical role of the nitric oxide/reactive oxygen species balance in endothelial progenitor dysfunction. Antioxid Redox Signal. (2011) 15:933–48. 10.1089/ars.2010.3502 20712407 PMC3135185

[108] Islam S Bostrom KI Di Carlo D Simmons CA Tintut Y Yao Y. The mechanobiology of endothelial-to-mesenchymal transition in cardiovascular disease. Front Physiol. (2021) 12:734215. 10.3389/fphys.2021.734215 34566697 PMC8458763

[109] Moonen JR Krenning G Brinker MG Koerts JA van Luyn MJ Harmsen MC. Endothelial progenitor cells give rise to pro-angiogenic smooth muscle-like progeny. Cardiovasc Res. (2010) 86:506–15. 10.1093/cvr/cvq012 20083576

[110] Sanchez-Duffhues G Garcia de Vinuesa A Ten Dijke P. Endothelial-to-mesenchymal transition in cardiovascular diseases: developmental signaling pathways gone awry. Dev Dyn. (2018) 247:492–508. 10.1002/dvdy.24589 28891150

[111] Ding H Yao J Xie H Wang C Chen J Wei K. MicroRNA-195-5p downregulation inhibits endothelial mesenchymal transition and myocardial fibrosis in diabetic cardiomyopathy by targeting Smad7 and inhibiting transforming growth factor beta 1-Smads-Snail pathway. Front Physiol. (2021) 12:709123. 10.3389/fphys.2021.709123 34658906 PMC8514870

[112] Salybekov AA Kunikeyev AD Kobayashi S Asahara T. Latest advances in endothelial progenitor cell-derived extracellular vesicles translation to the clinic. Front Cardiovasc Med. (2021) 8:734562. 10.3389/fcvm.2021.734562 34671654 PMC8520929

[113] Shudo Y Cohen JE Macarthur JW Atluri P Hsiao PF Yang EC. Spatially oriented, temporally sequential smooth muscle cell-endothelial progenitor cell bi-level cell sheet neovascularizes ischemic myocardium. Circulation. (2013) 128:S59–68. 10.1161/CIRCULATIONAHA.112.000293 24030422 PMC4111240

[114] Shudo Y Goldstone AB Cohen JE Patel JB Hopkins MS Steele AN. Layered smooth muscle cell-endothelial progenitor cell sheets derived from the bone marrow augment postinfarction ventricular function. J Thorac Cardiovasc Surg. (2017) 154:955–63. 10.1016/j.jtcvs.2017.04.081 28651946 PMC5947323

[115] Eschenhagen T Bolli R Braun T Field LJ Fleischmann BK Frisen J. Cardiomyocyte regeneration: a consensus statement. Circulation. (2017) 136:680–6. 10.1161/CIRCULATIONAHA.117.029343 28684531 PMC5557671

[116] Yeh JN Yang RR Wallace CG Huang CR Chu YC Yip HK. Impact of one versus two consecutive doses of endothelial cells (EPCs) and EPCs-derived condition medium on protecting myocardium from Acute Ischemia-Reperfusion injury in rat. Cell Transplant. (2021) 30:9636897211007049. 10.1177/09636897211007049 33975445 PMC8120601

[117] Xue Y Zhou B Wu J Miao G Li K Li S. Transplantation of endothelial progenitor cells in the treatment of coronary artery microembolism in rats. Cell Transplant. (2020) 29:963689720912688. 10.1177/0963689720912688 32233803 PMC7444210

[118] Aurora AB Mahmoud AI Luo X Johnson BA van Rooij E Matsuzaki S. MicroRNA-214 protects the mouse heart from ischemic injury by controlling Ca^2^? overload and cell death. J Clin Invest. (2012) 122:1222–32. 10.1172/JCI59327 22426211 PMC3314458

[119] Souders CA Bowers SL Baudino TA. Cardiac fibroblast: the renaissance cell. Circ Res. (2009) 105:1164–76. 10.1161/CIRCRESAHA.109.209809 19959782 PMC3345531

[120] Pardali E Sanchez-Duffhues G Gomez-Puerto MC Ten Dijke P. TGF-beta-induced endothelial-mesenchymal transition in fibrotic diseases. Int J Mol Sci. (2017) 18:2157. 10.3390/ijms18102157 29039786 PMC5666838

[121] Ubil E Duan J Pillai IC Rosa-Garrido M Wu Y Bargiacchi F. Mesenchymal-endothelial transition contributes to cardiac neovascularization. Nature. (2014) 514:585–90. 10.1038/nature13839 25317562 PMC4214889

[122] Ke X Yang D Liang J Wang X Wu S Wang X. Human endothelial progenitor cell-derived exosomes increase proliferation and angiogenesis in cardiac fibroblasts by promoting the mesenchymal-endothelial transition and reducing high mobility group box 1 protein B1 expression. DNA Cell Biol. (2017) 36:1018–28. 10.1089/dna.2017.3836 28920705

[123] Huang Y Chen L Feng Z Chen W Yan S Yang R. EPC-Derived Exosomal miR-1246 and miR-1290 regulate phenotypic changes of fibroblasts to endothelial cells to exert protective effects on myocardial infarction by targeting ELF5 and SP1. Front Cell Dev Biol. (2021) 9:647763. 10.3389/fcell.2021.647763 34055778 PMC8155602

[124] Ke X Yang R Wu F Wang X Liang J Hu X. Exosomal miR-218-5p/miR-363-3p from endothelial progenitor cells ameliorate myocardial infarction by targeting the p53/JMY signaling pathway. Oxid Med Cell Longev. (2021) 2021:5529430. 10.1155/2021/5529430 34326916 PMC8302385

[125] Kasai-Brunswick TH Carvalho AB Campos de Carvalho AC. Stem cell therapies in cardiac diseases: current status and future possibilities. World J Stem Cells. (2021) 13:1231–47. 10.4252/wjsc.v13.i9.1231 34630860 PMC8474720

[126] Balbi C Lodder K Costa A Moimas S Moccia F van Herwaarden T. Reactivating endogenous mechanisms of cardiac regeneration via paracrine boosting using the human amniotic fluid stem cell secretome. Int J Cardiol. (2019) 287:87–95. 10.1016/j.ijcard.2019.04.011 30987834

[127] Deutsch MA Brunner S Grabmaier U David R Ott I Huber BC. Cardioprotective potential of human endothelial-colony forming cells from diabetic and nondiabetic donors. Cells. (2020) 9:588. 10.3390/cells9030588 32131432 PMC7140510

[128] Poh KK Lee PSS Djohan AH Galupo MJ Songco GG Yeo TC. Transplantation of endothelial progenitor cells in obese diabetic rats following myocardial infarction: role of thymosin beta-4. Cells. (2020) 9:949. 10.3390/cells9040949 32290541 PMC7226991

[129] Williams AR Hare JM. Mesenchymal stem cells: biology, pathophysiology, translational findings, and therapeutic implications for cardiac disease. Circ Res. (2011) 109:923–40. 10.1161/CIRCRESAHA.111.243147 21960725 PMC3604746

[130] Kim SW Jin HL Kang SM Kim S Yoo KJ Jang Y. Therapeutic effects of late outgrowth endothelial progenitor cells or mesenchymal stem cells derived from human umbilical cord blood on infarct repair. Int J Cardiol. (2016) 203:498–507. 10.1016/j.ijcard.2015.10.110 26551883 PMC4688096

[131] Aguirre A Planell JA Engel E. Dynamics of bone marrow-derived endothelial progenitor cell/mesenchymal stem cell interaction in co-culture and its implications in angiogenesis. Biochem Biophys Res Commun. (2010) 400:284–91. 10.1016/j.bbrc.2010.08.073 20732306

[132] Popescu S Preda MB Marinescu CI Simionescu M Burlacu A. Dual stem cell therapy improves the myocardial recovery post-infarction through reciprocal modulation of cell functions. Int J Mol Sci. (2021) 22:5631. 10.3390/ijms22115631 34073327 PMC8199446

[133] Burlacu A Grigorescu G Rosca AM Preda MB Simionescu M. Factors secreted by mesenchymal stem cells and endothelial progenitor cells have complementary effects on angiogenesis *in vitro*. Stem Cells Dev. (2013) 22:643–53. 10.1089/scd.2012.0273 22947186 PMC3564466

[134] Zhao YH Yuan B Chen J Feng DH Zhao B Qin C. Endothelial progenitor cells: therapeutic perspective for ischemic stroke. CNS Neurosci Ther. (2013) 19:67–75. 10.1111/cns.12040 23230897 PMC4043291

[135] António N Fernandes R Rodriguez-Losada N Jiménez-Navarro MF Paiva A de Teresa Galván E. Stimulation of endothelial progenitor cells: a new putative effect of several cardiovascular drugs. Eur J Clin Pharmacol. (2010) 66:219–30. 10.1007/s00228-009-0764-y 20012029

[136] Klomp M Beijk MA de Winter RJ. Genous endothelial progenitor cell-capturing stent system: a novel stent technology. Expert Rev Med Devices. (2009) 6:365–75. 10.1586/erd.09.16 19572791

[137] Kou F Zhu C Wan H Xue F Wang J Xiang L. Endothelial progenitor cells as the target for cardiovascular disease prediction, personalized prevention, and treatments: progressing beyond the state-of-the-art. EPMA J. (2020) 11:629–43. 10.1007/s13167-020-00223-0 33240451 PMC7680476

[138] Jimenez-Quevedo P Gonzalez-Ferrer JJ Sabate M Garcia-Moll X Delgado-Bolton R Llorente L. Selected CD133? progenitor cells to promote angiogenesis in patients with refractory angina: final results of the PROGENITOR randomized trial. Circ Res. (2014) 115:950–60. 10.1161/CIRCRESAHA.115.303463 25231095

[139] Stamm C Kleine HD Choi YH Dunkelmann S Lauffs JA Lorenzen B. Intramyocardial delivery of CD133+ bone marrow cells and coronary artery bypass grafting for chronic ischemic heart disease: safety and efficacy studies. J Thorac Cardiovasc Surg. (2007) 133:717–25. 10.1016/j.jtcvs.2006.08.077 17320570

[140] De Rosa S Seeger FH Honold J Fischer-Rasokat U Lehmann R Fichtlscherer S. Procedural safety and predictors of acute outcome of intracoronary administration of progenitor cells in 775 consecutive procedures performed for acute myocardial infarction or chronic heart failure. Circ Cardiovasc Interv. (2013) 6:44–51. 10.1161/CIRCINTERVENTIONS.112.971705 23362308

[141] Miyagawa S Roth M Saito A Sawa Y Kostin S. Tissue-engineered cardiac constructs for cardiac repair. Ann Thorac Surg. (2011) 91:320–9. 10.1016/j.athoracsur.2010.09.080 21172551

[142] Thej C Kishore R. Unfathomed nanomessages to the heart: translational implications of stem cell-derived, progenitor cell exosomes in cardiac repair and regeneration. Cells. (2021) 10:1811. 10.3390/cells10071811 34359980 PMC8307947

[143] Chen CW Wang LL Zaman S Gordon J Arisi MF Venkataraman CM. Sustained release of endothelial progenitor cell-derived extracellular vesicles from shear-thinning hydrogels improves angiogenesis and promotes function after myocardial infarction. Cardiovasc Res. (2018) 114:1029–40. 10.1093/cvr/cvy067 29566124 PMC5967544

[144] O'Neill CL O'Doherty MT Wilson SE Rana AA Hirst CE Stitt AW. Therapeutic revascularisation of ischaemic tissue: the opportunities and challenges for therapy using vascular stem/progenitor cells. Stem Cell Res Ther. (2012) 3:31. 10.1186/scrt122 22897941 PMC3580469

[145] Keighron C Lyons CJ Creane M O'Brien T Liew A. Recent advances in endothelial progenitor cells toward their use in clinical translation. Front Med. (2018) 5:354. 10.3389/fmed.2018.00354 30619864 PMC6305310

[146] Prasain N Lee MR Vemula S Meador JL Yoshimoto M Ferkowicz MJ. Differentiation of human pluripotent stem cells to cells similar to cord-blood endothelial colony-forming cells. Nat Biotechnol. (2014) 32:1151–7. 10.1038/nbt.3048 25306246 PMC4318247

[147] Peyter AC Armengaud JB Guillot E Yzydorczyk C. Endothelial progenitor cells dysfunctions and cardiometabolic disorders: from mechanisms to therapeutic approaches. Int J Mol Sci. (2021) 22:6667. 10.3390/ijms22136667 34206404 PMC8267891

[148] Eisen A Leshem-Lev D Yavin H Orvin K Mager A Rechavia E. Effect of high dose statin pretreatment on endothelial progenitor cells after percutaneous coronary intervention (HIPOCRATES Study). Cardiovasc Drugs Ther. (2015) 29:129–35. 10.1007/s10557-015-6575-8 25712416

[149] Zhu J Song J Yu L Zheng H Zhou B Weng S. Safety and efficacy of autologous thymosin β4 pre-treated endothelial progenitor cell transplantation in patients with acute ST segment elevation myocardial infarction: a pilot study. Cytotherapy. (2016) 18:1037–42. 10.1016/j.jcyt.2016.05.006 27288307

